# Dynamic functional connectivity analysis with temporal convolutional network for attention deficit/hyperactivity disorder identification

**DOI:** 10.3389/fnins.2023.1322967

**Published:** 2023-12-11

**Authors:** Mingliang Wang, Lingyao Zhu, Xizhi Li, Yong Pan, Long Li

**Affiliations:** ^1^School of Computer and Software, Nanjing University of Information Science and Technology, Nanjing, China; ^2^Nanjing Xinda Institute of Safety and Emergency Management, Nanjing, China; ^3^MIIT Key Laboratory of Pattern Analysis and Machine Intelligence, Nanjing University of Aeronautics and Astronautics, Nanjing, China; ^4^School of Accounting, Nanjing University of Finance and Economics, Nanjing, China; ^5^Taian Tumor Prevention and Treatment Hospital, Taian, China

**Keywords:** functional connectivity, temporal dependence, dynamics characteristics, attention deficit/hyperactivity disorder, temporal convolutional network

## Abstract

**Introduction:**

Dynamic functional connectivity (dFC), which can capture the abnormality of brain activity over time in resting-state functional magnetic resonance imaging (rs-fMRI) data, has a natural advantage in revealing the abnormal mechanism of brain activity in patients with Attention Deficit/Hyperactivity Disorder (ADHD). Several deep learning methods have been proposed to learn dynamic changes from rs-fMRI for FC analysis, and achieved superior performance than those using static FC. However, most existing methods only consider dependencies of two adjacent timestamps, which is limited when the change is related to the course of many timestamps.

**Methods:**

In this paper, we propose a novel Temporal Dependence neural Network (TDNet) for FC representation learning and temporal-dependence relationship tracking from rs-fMRI time series for automated ADHD identification. Specifically, we first partition rs-fMRI time series into a sequence of consecutive and non-overlapping segments. For each segment, we design an FC generation module to learn more discriminative representations to construct dynamic FCs. Then, we employ the Temporal Convolutional Network (TCN) to efficiently capture long-range temporal patterns with dilated convolutions, followed by three fully connected layers for disease prediction.

**Results:**

As the results, we found that considering the dynamic characteristics of rs-fMRI time series data is beneficial to obtain better diagnostic performance. In addition, dynamic FC networks generated in a data-driven manner are more informative than those constructed by Pearson correlation coefficients.

**Discussion:**

We validate the effectiveness of the proposed approach through extensive experiments on the public ADHD-200 database, and the results demonstrate the superiority of the proposed model over state-of-the-art methods in ADHD identification.

## 1 Introduction

Attention deficit/hyperactivity disorder (ADHD) is one of the most common neurodevelopmental disorders that typically appears in early childhood. It is characterized by significant symptoms of inattention, impulsivity, and hyperactivity, resulting in substantial functional impairment in at least two settings (e.g., social, occupational, and/or academic activities) (American Psychiatric Association et al., 2013). It is estimated that ADHD affects approximately 7.2% of people worldwide (Thomas et al., 2015). The current diagnosis of ADHD mostly relies on behavior assessment and clinical measures to quantify the severity of the disorder (Sayal et al., 2018; Chan et al., 2023), making it a challenging task due to the complexity of its pathological mechanisms and clinical symptoms (Usami, 2016). Therefore, the advent of any computer-aided diagnosis method that supports an objective and quantitative method to identify ADHD automatically is highly desirable.

Resting-state functional magnetic resonance imaging (rs-fMRI), a non-invasive neuroimaging technique that measures spontaneous fluctuations in blood oxygen level dependent (BOLD) signal at rest, has been widely used to study brain function in humans (Lee et al., 2013; Cortese et al., 2021; Wang et al., 2023). Functional connectivity (FC) derived from rs-fMRI is able to characterize brain function abnormality and thus has been widely used for diagnosis of psychiatric diseases, such as schizophrenia, autism spectrum disorders (ASD), and attention deficit/hyperactivity disorders (ADHD) (Du et al., 2018; Wang et al., 2019c; Canario et al., 2021). In the field of fMRI analysis, it is typically assumed that the brain FC is stationary over the whole scanning process (usually several minutes). In fact, increasing evidence suggests that the FCs change considerably on a short time scale (several seconds) (Zhang et al., 2016; Jie et al., 2018; Ding et al., 2022; Huang et al., 2023), and the static FC analysis cannot sufficiently perceive these dynamic changes. The sliding window approach is the commonly used technique to quantify dynamic FC (dFC). According to this method, BOLD time series extracted from each subject are first partitioned into multiple overlapping or non-overlapping segments using fixed-size sliding windows, and then the FC network based on each segment is constructed for subsequent analysis.

Existing methods for dFC analysis based on sliding windows can be roughly categorized into two categories: (1) traditional machine learning methods and (2) deep learning methods. In the first category, low-level measures (i.e., clustering coefficients) of FCs are first extracted as new representations of the data, and then the corresponding classifier (i.e., support vector machine, SVM) is trained for final prediction (Wee et al., 2016; Jie et al., 2018; Wang et al., 2021). For example, Wee et al. (2016) proposed to use the fused multiple group LASSO algorithm to simultaneously generate dFC networks for these sub-segments. Then, clustering coefficients are calculated from each generated FC network. Finally, the concatenated clustering coefficients of all these segments are used to train an SVM classifier for disease diagnosis. Jie et al. (2018) first constructed dynamic FCs from each segment and then extracted temporal and spatial variabilities from these FCs as features. Finally, the manifold regularized multi-task feature learning and multi-kernel learning techniques are used to integrate these features for disease prediction. Luo et al. (2023) proposed to calculate temporal microstate dynamics and spectral power features to analyze group differences between ADHD and normal controls (NCs), as well as its subtypes. These studies show that taking dynamic properties into consideration helps improve the performance of disease diagnosis, and the discovered changes in FCs may be potential biomarkers to distinguish patients from normal controls. However, existing methods based on traditional machine learning usually rely on handcrafted features to learn models for subsequent classification/prediction tasks, which may lead to sub-optimal performance.

The second category (i.e., deep learning methods) has been widely used in dFC analysis due to its powerful learning ability (Wang et al., 2019b; Li et al., 2020; Cao et al., 2022). Different from traditional machine learning methods, deep learning methods usually learn high-level features from dFC networks in a data-driven manner, which can effectively improve learning performance. For example, Wang et al. (2019b) proposed a Spatial-Temporal convolutional-recurrent neural Network (STNet) for Alzheimer's disease progression prediction using rs-fMRI time series. Specifically, a convolutional component was employed to construct the FC within each time-series segment. Then, the long short-term memory (LSTM) units were used to model the temporal dynamics patterns of these successive FCs, followed by a fully-connected layer to perform disease progression prediction. Lin et al. (2022) developed a Convolutional Recurrent Neural Network (CRNN) for dynamic FCs analysis for automated brain disease diagnosis. In this method, a sequence of pre-constructed FC networks is input into three convolutional layers to extract temporal features, and an LSTM layer is used to capture temporal information for multiple time segments, followed by three fully connected layers for brain disease classification. To take advantage of spatiotemporal information of fMRI data, Yan et al. (2019) designed a Multi-scale RNN framework for schizophrenia classification. Specifically, stacked convolution layers were used to extract different scale features, followed by a two-layer stacked Gated Recurrent Unit (GRU) for dynamic information mining. Zhao et al. (2022) designed a hybrid deep learning framework, including a convolutional recurrent neural network with attention module (C-RNN^*AM*^) and a deep neural network (DNN). Specifically, C-RNN^*AM*^ was used to extract temporal dynamic dependencies with an attention module to automatically learn discriminative knowledge from time courses, while DNN was applied to learn FC patterns with layer-wise relevance propagation. Then, the two outputs were concatenated and fed to logistic regression for final prediction.

**Figure 1:**
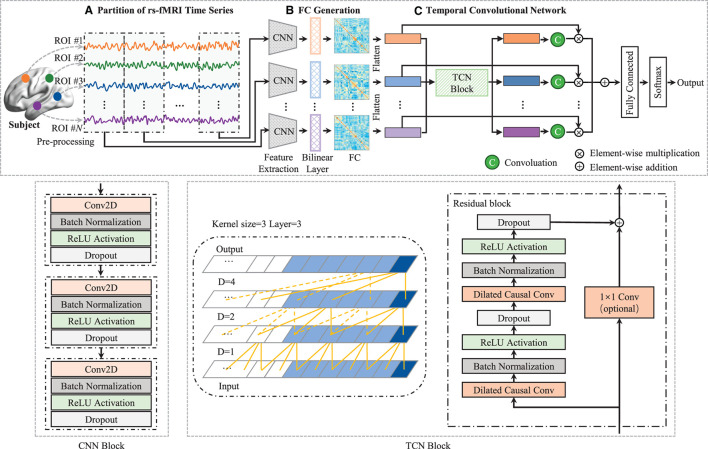
Architecture of TDNet, including three components: **(A)** partitioning rs-fMRI time series via non-overlapping sliding windows, **(B)** a FC generation module (with three cascaded convolutional layers and a bilinear operation layer) to construct functional connectivity within each time window, and **(C)** a temporal convolutional network to capture temporal dynamics across all the time windows. With the output of the temporal convolutional network, a convolution is used to obtain these probabilistic values to fuse these time windows, followed by three fully-connected layers and a softmax activation for final disease identification. The left in TCN block is a dilated causal convolution with dilation factors *D* = 1, 2, 4 and filter size *k* = 3. The right is a TCN residual block. A 1 × 1 convolution is added when residual input and output have different dimensions.

Although existing deep learning methods have advanced the dFC analysis from learning efficiency and classification accuracy perspectives, they only can capture the dependencies of adjacent timestamps. Previous studies (Wang et al., 2019b; Lin et al., 2022) usually employed recurrent neural network (RNN) with LSTM or GRU. To capture temporal dependency for dFC analysis. While in these methods, the latent state at each time segment *t*, is only a function of the data at *t* and the hidden state and memory at *t*−1. This is limiting when the dynamics at time segment *t* are related to the course of many timestamps (Lea et al., 2017). To this end, in this paper, we propose a novel dynamic FC analysis model called Temporal Dependence neural Network (TDNet), which can capture temporal-dependence relationships from rs-fMRI time series for automated ADHD diagnosis. Figure 1 shows an overview of the proposed TDNet model. Specifically, we first partition rs-fMRI time series into a sequence of consecutive and non-overlapping segments using fixed-size sliding windows. Then, we utilize FC generation module, which consists of three convolution layers and a bilinear operation layer, to learn the FC for each segment. To capture the temporal dependencies across multiple segments, a temporal convolutional network (TCN) using dilated causal convolutions and residual blocks is employed in the TDNet model. Finally, we apply a convolution operation over the output of TCN to get the probabilistic values to fuse dFCs, followed by three fully-connected layers and a softmax activation for final prediction. Extensive experimental results on the ADHD-200 dataset show the effectiveness of our model in capturing temporal dynamic patterns and producing high disease diagnosis accuracy.

## 2 Materials and methods

In this section, we describe the materials used in this study, as well as the proposed method for dynamic functional connectivity analysis with rs-fMRI time series.

### 2.1 Materials

#### 2.1.1 Data acquisition

In this study, we conduct experiments on the publicly available ADHD-200 dataset to demonstrate the effectiveness of our TDNet model. The ADHD-200 dataset totally collected 973 subjects from eight different imaging sites, including 362 ADHD patients, 585 normal controls (NCs), and 26 undiagnosed subjects. The data is available from the NeuroImaging Tools & Resource Collaboratory (NITRC) website.^1^ Each participant's data is composed of a resting state functional MRI scan, a structural MRI scan, and the corresponding phenotypic information. More details about the scan procedures and parameters are also described on the NITRC website.^2^ Note that ADHD patients within the dataset are subdivided into three subtypes, including ADHD-Combined, ADHD-Hyperactive/Impulsive, and ADHD-Inattentive. For simplicity, we will ignore subtypes in the binary classification task and label all subtypes as 1. In the ADHD-200 Global Competition, the data was divided into a training set and a test set, and the corresponding numbers of subjects were 768 and 197, respectively. In this paper, we also follow this division in our experiments. Due to the labels of 26 subjects in the test set have not been released, they were not included in our performance evaluation. Besides, subjects from the Pitt and Washu imaging sites were also discarded in our study because they only contained NC subjects in the training set. Thus, a total of 782 subjects were used in this study, including 620 ADHD patients and 162 NCs. The detailed demographic information of involved subjects and data partition for experiments are provided in Table 1.

**Table 1:** Demographic information of the studied subjects from the ADHD database.

**Item**	**KKI**	**NI**	**NYU**	**OHSU**	**PKU**	**Total**
**Training dataset**						
Number	83	48	216	79	194	620
NC	61	23	98	42	116	340
ADHD	22	25	118	37	78	280
Gender(M/F)	46/37	31/17	140/76	43/36	144/50	404/216
**Item**	**KKI**	**NI**	**NYU**	**OHSU**	**PKU**	**Total**
**Test dataset**						
Number	11	25	41	34	51	162
NC	8	14	12	28	27	94
ADHD	3	11	29	6	24	73
Gender(M/F)	10/1	12/13	28/13	17/17	32/19	99/63

NC, normal control; M/F, male/female.

#### 2.1.2 Data preprocessing

All the rs-fMRI data used in this study are processed by using the Athena pipeline.^3^ Specifically, the first four volumes are removed to allow for magnetization equilibrium, and then the remaining volumes are processed by the following procedures, including slice timing correction, head motion correction, Montreal Neurological Institute (MNI) space normalization, and re-sampling at 4 × 4 × 4*mm*^3^ resolution. After that, mean white matter (WM) and cerebrospinal fluid (CSF) signals, six head motion parameters, and a third-order polynomial were included in a voxelwise nuisance regression model to eliminate time series variations caused by physiological noise, head motion, and scanner drifts. The rs-fMRI data were then further spatially smoothed with a 6*mm* full-width-at-half-maximum (FWHM) Gaussian kernel and temporally filtered to preserve the signals of 0.009 − 0.08*Hz*. Finally, the time series of 116 pre-defined regions-of-interest (ROIs) are extracted from the preprocessed data using the automated anatomical labeling (AAL) atlas (Tzourio-Mazoyer et al., 2002).

### 2.2 Methods

As illustrated in Figure 1, the proposed TDNet consists of three modules: (a) an rs-fMRI time series partition module, (b) a FC generation module, and (c) a temporal convolutional network module. Given a set of labeled fMRI time series, $D=\left({\text{X}}^{\left(i\right)},y^{\left(i\right)}\right)$, ${\text{X}}^{\left(i\right)}={\left({\text{x}}_{1}^{\left(i\right)},\cdots \;,{\text{x}}_{N}^{\left(i\right)}\right)}^{T}\in {\mathbb{R}}^{N\times M}$ represents the *i*-th subject, which contains *N* time series (*N* = 116). Here, ${\text{x}}_{n}^{\left(i\right)}\in {\mathbb{R}}^{M}$ (*n* = 1⋯ , *N*) denotes the *n*-th time series of *i*-th subject with *M* successive time points. In addition, *y*^(*i*)^ ∈ {−1, 1} denotes the class label of **X**^(*i*)^. Specifically, *y*^(*i*)^ = 1 denotes the subject is an ADHD patient, while *y*^(*i*)^ = −1 represents the subject belonging to the normal control (NC) group. The proposed TDNet takes preprocessed time series signals (i.e., **X**) from rs-fMRI data as the input, which aims to model dynamic characteristics of such data for disease diagnosis.

#### 2.2.1 Partition of rs-fMRI time series

We first employ the sliding window strategy to partition all rs-fMRI time series into *T* non-overlapping windows with a fixed window size *L* to preserve the temporal variability. Specifically, we set the window size *L* as 20 time points, by which $\text{S}={\left\{{\text{X}}^{t}\in {\mathbb{R}}^{N\times L}\right\}}_{t=1}^{T}$ denote the resulting time series segments. The lengths of extracted time series are 119, 257, 171, 74, and 231 repetition time (TR) for the KKI, NI, NYU, OHSU, and PKU datasets, respectively, and the corresponding TR is 2.5, 1.96, 2, 2.5, and 2s. Due to the scanning time of each site is different, we obtain *T* = 5, *T* = 12, *T* = 8, *T* = 3, and *T* = 11 time-series segments for these sites, respectively. It is worth noting that we discard windows with time points less than 20 in the experiment. The reason for choosing such window length is that window sizes around 30-60s can provide a robust estimation of the dynamic fluctuations in rs-fMRI data (Zhang et al., 2016; Wang et al., 2021). For each subject, the time-series segments **S** will be considered as the input of the proposed network (as introduced below).

#### 2.2.2 FC generation module

In this module, a convolutional neural network (CNN) block is used to extract features from each brain region's time-series signals at each time segment for learning high-level representations. As depicted in the middle of Figure 1, the CNN block consists of 3 convolutional (Conv) layers, and the *k*-th layer can be denoted by ${\text{F}}_{k}\in {\mathbb{R}}^{f_{k}\times N\times d_{k}}$, where *f*_*k*_ is the number of convolutional filters in the *k*-th layer, *N* is the number of ROIs, and *d*_*k*_ is the dimension of the extracted region features, respectively. Each layer consists of convolutional, batch normalization (BN), rectified linear unit (ReLU), and dropout operations. We define the collection of filters in each layer as ${\text{W}}_{k}\in {\mathbb{R}}^{f_{k}\times 1\times L_{k}}$, and denote the bias vectors as ${\text{b}}_{k}\in {\mathbb{R}}^{f_{k}}$. Given the time series from the previous preprocessing, ${\text{F}}_{k-1}^{t}$ (${\text{F}}_{0}^{t}\in {\mathbb{R}}^{1\times N\times L}$), we compute activations ${\text{F}}_{k}^{t}$ as follows:


(1) $$\begin{array}{l} {\text{F}}_{k}^{t}=ReLU\left(BN\left(\left({\text{W}}_{k}*{\text{F}}_{k-1}^{t}+{\text{b}}_{k}\right)\right)\right), \end{array}$$

where * is the convolution operator. Based on the encoded time-series feature **F**^*t*^ of *t*-th time series segment, a bilinear operation is employed to model dependencies between pairs of ROIs. Specifically, for each subject, we calculate the inner product between each pair of brain regions to generate a functional connectivity matrix **A**^*t*^ as:


(2) $$\begin{array}{l} {\text{A}}^{t}={\text{F}}^{t}{\left({\text{F}}^{t}\right)}^{⊤}, \end{array}$$

where **A**^*t*^ ∈ ℝ^1×*N*×*N*^ and each element ${\text{A}}_{ij}^{t}=\overset{d_{3}}{\underset{q=1}{\sum }}{\text{F}}_{iq}^{t}{\text{F}}_{jq}^{t}$ measures the degree of second-order dependency between two brain regions. A large value of ${\text{A}}_{ij}^{t}$ indicates the *i*-th and *j*-th ROIs are highly related.

#### 2.2.3 Temporal convolutional network module

In this module, a temporal convolutional network (TCN) with dilated causal convolutions and residual blocks is employed to model temporal dependencies for DFC analysis. Specifically, the TCN uses residual blocks as the building blocks (Bai et al., 2018). One residual block of the TCN architecture can be seen in the bottom right of Figure 1, which is composed of two sets of dilated causal convolution layers with the same dilation factor, followed by batch normalization, ReLU activation, and dropout layers. The dilated causal convolutional layer is the core network layer of the TCN, which consists of two parts: causal convolution (Long et al., 2015) and dilated convolution (Oord et al., 2016). The causal convolutions ensure that the network produces an output of the same length as the input, and there is no future information leakage. The dilated convolutions help the network to increase its receptive field without the need to increase the number of parameters by increasing the number of layers or the kernel size. By increasing the dilation factor, the receptive field also grows exponentially with the number of layers. In the TCN block (see in the right of Figure 1), the residual connection combines the input and the output feature maps, and if the depth of the input and output is different, a 1 × 1 convolution is used. The set of operations at each layer can be mathematically described as follows:


(3) $$\begin{array}{l} \begin{array}{l} {\tilde{\text{H}}}_{q}=ReLU\left({\text{W}}_{2}*ReLU\left({\text{W}}_{1}*{\text{H}}_{q-1}+b_{1}\right)+b_{2}\right),\\ {\text{H}}_{q}={\text{H}}_{q-1}+{\text{W}}_{3}*{\tilde{\text{H}}}_{q}+b_{3}, \end{array} \end{array}$$

where **H**_*q*_ denotes the output of *q*-th (*q* ∈ [1, ⋯ , *Q*]) residual block, * is the convolution operator, ${\text{W}}_{1},{\text{W}}_{2}\in {\mathbb{R}}^{3\times f_{q}}$ are the weights of the dilated convolution filters with kernel size 3 and *f*_*q*_ is the number of convolution filters, ${\text{W}}_{3}\in {\mathbb{R}}^{1\times f_{q}}$ are the weights of a 1 × 1 convolution, and $b_{1},b_{2},b_{3}\in {\mathbb{R}}^{f_{q}}$ are bias vectors. These operations are depicted in Figure 1. Note that although TCN only has 1D dilated causal convolutions, they are still capable of processing 2D feature maps by considering the second dimension as the depth dimension. For each generated FC (i.e., **A**^*t*^, *t* ∈ [1, ⋯ , *T*]), we first remove the upper triangle and the diagonal elements and convert the remaining elements into a *N*(*N*−1)/2-dimensional vectorized representation. Then, we stack the vectorized representation of these FCs into a longitudinally ordered sequence, denoted as **H** ∈ ℝ^*T*×*N*(*N* − 1)/2^, and treat it as the input to the TCN.

By stacking several residual blocks, the receptive field of the proposed TCN is determined through the following:


(4) $$\begin{array}{l} ReceptiveField=1+2\left(2^{Q}-1\right)\left(K-1\right), \end{array}$$

where *Q* is the number of residual blocks and *K* is the kernel size. To fuse temporal dynamic patterns, we further apply a 1 × 1 convolution over the output of the last dilated convolution layer followed by a *tanh* activation,


(5) $$\begin{array}{l} \text{P}=tanh\left(\text{W}{\text{H}}_{Q}+\text{b}\right), \end{array}$$

where **P** ∈ ℝ^*T*^ denotes the contribution of these time-series segments for ADHD diagnosis, **H**_*Q*_ is the output of the last dilated convolution layer, **W** ∈ ℝ^1×*D*^ and **b** ∈ ℝ^*D*^ are the trainable weights and bias for the 1 × 1 convolution layer, *D* is the number of convolutional filters. After that, **P** is applied to obtain the fused dFCs representation **H**′, which can be defined as follows:


(6) $$\begin{array}{l} {\text{H}}^{\prime }=\text{P}\text{H}. \end{array}$$

Finally, the fused representation is fed to three fully-connected layers for classification.

#### 2.2.4 Implementation details

The TDNet model was implemented using Python based on the Keras package,^4^ and the model was trained on an NVIDIA GeForce GTX 2080 Ti GPU. In the CNN block, the number of convolutional filters for three convolutional layers was set as 4, 2, and 1, respectively, and the kernel size of these layers were 1 × 3, 1 × 3 and 1 × 1. In the TCN block, the number of convolution filters and residual blocks were both set to 3. Three fully-connected layers with 512, 128, and 2 neurons, respectively. The categorical_crossentropy was used as the loss function and the dropout rate of dropout layers was 0.3. The model is trained for 100 epochs with an Adam optimizer at recommended parameter settings and a batch size of 16.

## 3 Experiments

### 3.1 Experimental setup

We evaluate the proposed method using the classification accuracy on rs-fMRI data from five different sites (i.e., KKI, NI, NYU, OHSU, and PKU) from the ADHD database. We compare our TDNet with the following six deep learning methods, including two static methods, two dynamic methods, and two variants of our method. (1) Multilayer Perceptron (MLP): The MLP contains two hidden layers with 512, and 128 neurons on each layer for ADHD diagnosis; (2) Convolution Neural Network (CNN): The CNN consists of three convolution layers with kernel size 5 × 5, 5 × 5, and 3 × 3, respectively, and kernel(s) for these layers are 8, 4, and 1. Two fully connected layers with 512 and 128 neurons, and an output layer with 2 neurons are followed. (3) Long Short-Term memory (LSTM): The LSTM contains three stacked layers with 512, 256, and 2 neurons, respectively, followed by softmax activation; and (4) CNN-LSTM: A simple combination of CNN and LSTM models. Specifically, the same architecture as CNN model is employed, but without using fully-connected layers. The output of the CNN is fed to an LSTM model to model temporal information. The CNN-LSTM model uses the same parameters as CNN and LSTM. (5) TDNet-F: To evaluate the effectiveness of our generated FC networks, the TDNet-F method was designed to use pre-constructed dynamic FCs as the input data, while the remaining network architecture was the same as TDNet. (6) TDNet-T: As a variant of TDNet, TDNet-T was implemented without considering the temporal dynamics along timestamps. That is, we replaced the TCN module with three fully-connected layers with 512, 128, and 2 neurons, respectively.

### 3.2 Classification performance

The classification accuracy of the proposed TDNet and other competing methods are shown in Table 2. From Table 2, we can have three interesting observations. *First*, dynamic FC-based models (i.e., LSTM, CNN-LSTM, TDNet-F, TDNet-T, and TDNet) generally outperform methods based on static FC networks (i.e., MLP and CNN). For example, the highest average accuracy achieved by TDNet is 73.2%, which is higher than those of static FC-based methods. This demonstrates that considering the dynamic changes of rs-fMRI time series is beneficial to improving the diagnostic performance of ADHD. *Second*, the proposed TDNet achieves consistently better results than the other comparative methods. For example, the highest accuracy value is 66.2% achieved by CNN model, which is significantly lower than our proposed model. This indicates that the data-driven construction of dynamic FCs helps boost the learning performance of TDNet. *Third*, compared with the LSTM and CNN-LSTM methods, our TDNet can obtain better performance, which proves the advantage of mining sequential dynamic patterns from rs-fMRI time series. *Finally*, our TDNet achieves an improvement of 6.9% and 4.1% compared with the results yielded by its two variants TDNet-F and TDNet-T, respectively. These results show that both modules are useful in helping to boost the learning performance of TDNet.

**Table 2:** Classification accuracy achieved by different methods on five datasets with rs-fMRI time-series data.

**Method**	**Site**					**Average**
	**KKI**	**NI**	**NYU**	**OHSU**	**PKU**	
MLP	72.7	60.0	65.9	70.6	51.0	64.0
CNN	63.6	64.0	68.3	70.6	64.7	66.2
LSTM	72.7	56.0	61.0	67.7	53.0	62.1
CNN-LSTM	63.6	56.0	68.3	73.5	58.8	64.1
TDNet-F	72.7	64.0	68.3	67.7	58.8	66.3
TDNet-T	72.7	64.0	70.7	73.5	64.7	69.1
TDNet (Ours)	**81.8**	**68.0**	**73.2**	**76.5**	**66.7**	**73.2**

Best results are shown in bold.

In addition, we also compare our method with several state- of-the-art approaches using rs-fMRI data from ADHD-200 database for ADHD identification. Such experimental results can be found in Table 1 of the Supplementary material.

### 3.3 Constructed functional connectivity

**Figure 2:**
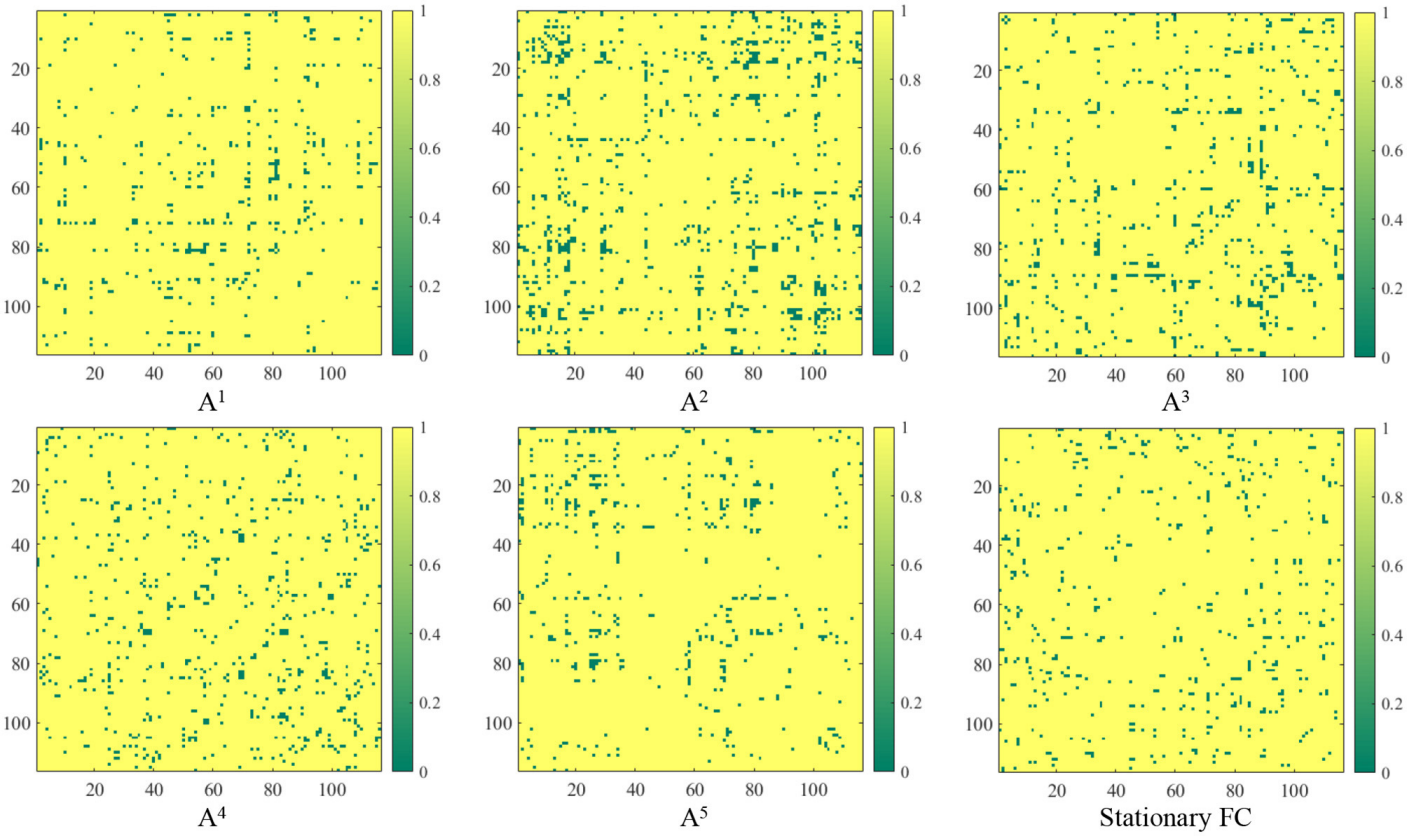
Group difference between the learned FC and the traditional “Stationary FC”. Here, *p*-values less than 0.05 between ADHD and NC groups are set to 0 (corresponding to the green parts in the figure). The term **A**^*t*^ (*t* = 1, 2, ⋯ , 5) corresponds to the group difference based on dynamic functional connectivities learned on *t*-th sliding window by the proposed FC generation module in TDNet.

In contrast to previous studies that rely on pre-defined FC networks (e.g., via Pearson's correlation) (Wee et al., 2016; Jie et al., 2018; Wang et al., 2019a, 2022), the proposed method can generate dynamic FC networks in a data-driven manner. We now investigated the FC networks constructed by the proposed TDNet in the KKI site. Specifically, the output of the FC generation module is a 4-dimensional tensor with the size of *Batch_size*×*T* × *N* × *N* (*T* = 5), denoting the dependency between a pair of ROIs for each subject in *T* time series segments. We measure the group difference between ADHD vs. NC using the standard *t*-test, with *p*-values shown in Figure 2. For comparison, in Figure 2, we also report the group difference of the stationary FC network (constructed by Pearson correlation coefficients between rs-fMRI time-series of any pair of ROIs). In Figure 2, the obtained *p*-values were binarized for clarity (i.e., set to 1 if the *p*-value is more than 0.05; and 0, otherwise).

From Figure 2, we can clearly see that there are significant differences in the discriminative information contained in different time series segments. Compared with other sub-segments, **A**^2^ contains relatively richer information, which indicates that considering temporal dynamic patterns can help improve learning performance. Additionally, the correlations learned by our TDNet are more informative than the static functional connectivity constructed by Pearson correlation coefficients, indicating that TDNet can identify more ADHD-related discriminative connectivities.

### 3.4 Identified discriminative brain regions

**Figure 3:**
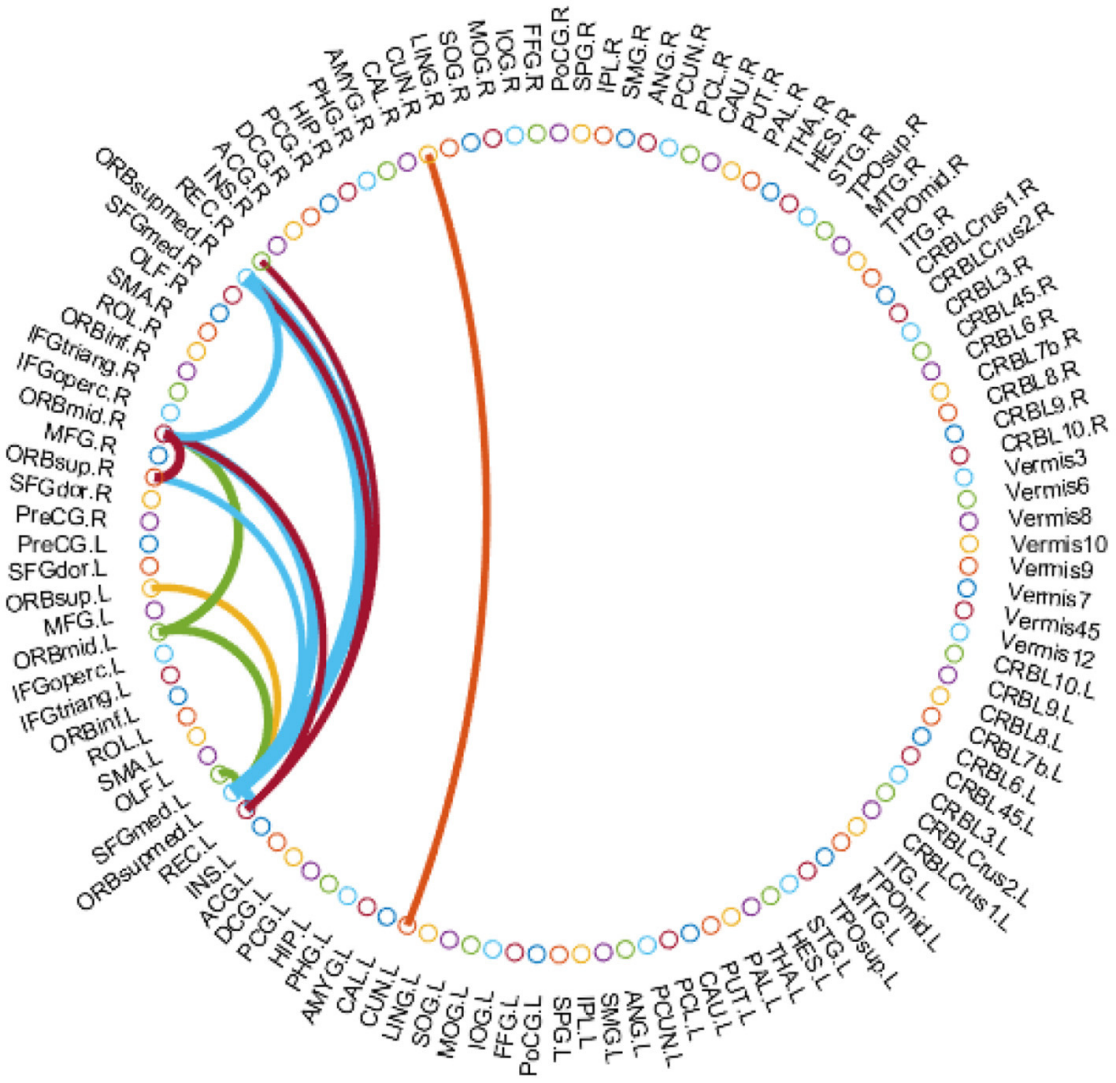
Top 15 brain functional connectivity patterns identified by our TDNet method in ADHD vs. NC classification on the KKI site.

It's meaningful to identify the discriminative brain regions that are associated with ADHD diagnosis. Here, we investigate the top 15 discriminative brain regions identified by the TDNet method on the KKI site. Since the identified brain connectivity patterns may be different in each segment, we use the cumulative absolute value in **A**^*t*^ as an indicator of its contribution to ADHD vs. NC classification. Hence, we calculate the cumulative absolute value of each element in **A**^*t*^ across all segments, and treat these values as the contribution indicator for subsequent classification tasks. We show the top 15 connectivity patterns identified by our TDNet in Figure 3, and further report the names of the corresponding brain ROIs in Table 3.

**Table 3:** Names of brain ROIs in the top 15 connectivity patterns identified by the proposed TDNet method.

**Index of pairwise ROI**	**ROI names**
26 & 25	ORBsupmed.R & ORBsupmed.L
25 & 10	ORBsupmed.L & ORBmid.R
27 & 25	REC.L & ORBsupmed.L
10 & 6	ORBmid.R & ORBsup.R
10 & 9	ORBmid.R & ORBmid.L
26 & 10	ORBsupmed.R & ORBmid.R
25 & 9	ORBsupmed.L & ORBmid.L
25 & 23	ORBsupmed.L & SFGmed.L
27 & 10	REC.L & ORBmid.R
25 & 6	ORBsupmed.L & ORBsup.R
46 & 45	CUN.R & CUN.L
27 & 26	REC.L & ORBsupmed.R
25 & 5	ORBsupmed.L & ORBsup.L
28 & 25	REC.R & ORBsupmed.L
28 & 27	REC.R & REC.L

ROI, region-of-interest.

From Figure 3 and Table 3, we can see that several brain regions, e.g., *Superior frontal gyrus, medial orbital* (ORBsupmed), *Middle frontal gyrus, orbital part* (ORBmid), and *Gyrus rectus* (REC), are selected frequently in the ADHD vs. NC classification task. These findings are consistent with previous studies (Joo et al., 2016; Itani et al., 2019; Sun et al., 2020). In addition, the selected brain regions such as *Superior frontal gyrus, orbital part* (ORBsup) and *Cuneus* (CUN) are also sensitive biomarkers for ADHD diagnosis, proven by previous studies (Sun et al., 2020; Lan et al., 2021). These results suggest that the identified brain regions by our method are reliable for ADHD analysis.

## 4 Discussion

### 4.1 Influence of different lengths of sliding windows

**Figure 4:**
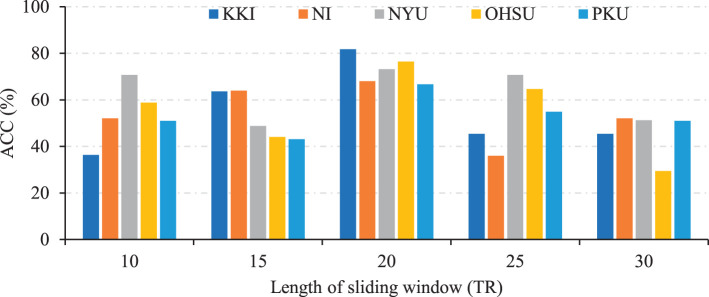
Results of the proposed TDNet method with respect to different lengths of sliding windows in ADHD vs. NC on different sites.

We also investigate the effects of different lengths of sliding windows on the TDNet method in terms of classification accuracy on five different sites. Specifically, we use a rectangle window, and vary the length of the sliding window to create time-series segments. The width of the sliding window is selected from the range of [10, 15, 20, 25, 30]×TR. In Figure 4, we report the classification accuracy achieved by our TDNet method under different lengths of sliding windows on ADHD vs. NC classification task. As can be seen from Figure 4, with the increases in the width of the sliding window, the classification accuracy fluctuates to a certain extent. When the width reaches 20×TR, our method achieves the best performance. These results suggest that it is reasonable to set the length of the sliding window to 20×TR.

### 4.2 Influence of Different Partition of Sliding Windows

**Figure 5:**
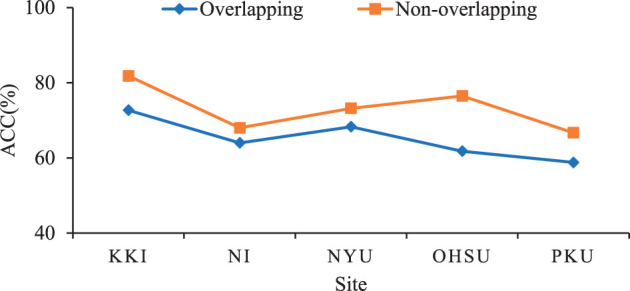
Results of the proposed TDNet method with respect to different partitions of sliding windows in ADHD vs. NC on different sites.

Furthermore, We investigate the influence of using overlapping sliding windows on the performance of the proposed method for ADHD diagnosis. Specifically, we set the window size *L* as 20 time points and the gap between two adjacent windows as 2×TR. This produces *T* = 6, *T* = 14, *T* = 9, *T* = 3, and *T* = 12 time series segments for these sites, respectively. The experimental results are shown in Figure 5. From Figure 5, we can clearly see that the classification performance obtained by our method using non-overlapping sliding windows on different sites is better than using overlapping ones. Therefore, using non-overlapping sliding windows to partition the time series is a more appropriate choice in experiments.

### 4.3 Limitations and future work

There are still several limitations that need to be considered in this study. *First*, we extract the time series signals from 116 ROIs based on the AAL atlas and generate the corresponding functional connectivity for ADHD diagnosis. Recent studies (Wu et al., 2019; Messé, 2020) have shown that FCs generated with different partitioning schemes can provide different connectivity patterns, which may contribute to more accurate brain disease diagnosis. It would be interesting to investigate the effect of different partitioning schemes on ADHD prediction. *Second*, we currently only use the rs-fMRI data to automatically identify ADHD in this study. In fact, different imaging modalities, such as structural MRI, can provide disease-related complementary information for disease diagnosis. The use of multi-modal information for brain disease analysis will be our future work. *Finally*, due to the different lengths of time series data in each imaging site, we are limited in the sample size that could be used simultaneously in this study. In future work, we will evaluate the proposed method on a larger dataset, such as Alzheimer's Disease Neuroimaging Initiative (ADNI) with rs-fMRI data.

## 5 Conclusion

In this paper, we propose a novel Temporal Dependence neural Network (TDNet) for automated diagnosis of ADHD using rs-fMRI time series. Specifically, we first partition rs-fMRI time series into multiple sub-segments using a non-overlapping sliding time window strategy. Then, an FC generation module is used to learn the FC for each segment and a temporal convolutional network module is employed to capture the temporal dependencies across these segments. Experimental results demonstrate that our model can achieve superior performance on the public ADHD-200 database compared to several state-of-the-art methods.

## Data availability statement

The original contributions presented in the study are included in the article/Supplementary material, further inquiries can be directed to the corresponding authors.

## Author contributions

MW: Conceptualization, Data curation, Formal analysis, Funding acquisition, Investigation, Methodology, Project administration, Resources, Supervision, Validation, Visualization, Writing—original draft, Writing—review & editing. LZ: Formal analysis, Methodology, Software, Validation, Writing—original draft. XL: Software, Validation, Writing—original draft. YP: Writing—review & editing. LL: Writing—review & editing.

## Footnotes

^1^ https://fcon_1000.projects.nitrc.org/indi/adhd200/

^2^ https://www.nitrc.org/plugins/mwiki/index.php/neurobureau:AthenaPipeline#Summary_Data

^3^ http://www.nitrc.org/plugins/mwiki/index.php/neurobureau:AthenaPipeline

^4^ https://github.com/fchollet/keras
