# Supplementary material for: Dynamic functional connectivity analysis with temporal convolutional network for attention deficit/hyperactivity disorder identification
Source: Front Neurosci. 2023 Dec 11;17:1322967. doi: 10.3389/fnins.2023.1322967 (PMC10750397; doi:10.3389/fnins.2023.1322967)
Supplement: Supplementary file 1 [file Data_Sheet_1.PDF]

Dynamic Functional Connectivity Analysis with Temporal Convolutional Network for Attention Deficit/Hyperactivity Disorder Identification – *Supplementary Materials*

Mingliang Wang [1,2,3], Lingyao Zhu [1] , Xizhi Li [1] , Yong Pan [4,*] and Long Li [5,*]

[1] *School of Computer and Software, Nanjing University of Information Science and Technology, Nanjing 210044, China*

[2] *Nanjing Xinda Institute of Safety and Emergency Management, Nanjing 210044, China*

[3] *MIIT Key Laboratory of Pattern Analysis and Machine Intelligence, Nanjing University of Aeronautics and Astronautics, Nanjing 211106, China*

[4] *School of Accounting, Nanjing University Of Finance and Economics, Nanjing 210023, China*

[5] *Taian Tumor Prevention and Treatment Hospital, Taian 271000, China*

Correspondence*:
Corresponding Author
panyong@nufe.edu.cn; wphone25@163.com;

In what follows, we compare our method with several state-of-the-art approaches using rs-fMRI data from ADHD-200 database for ADHD identification.

1 COMPARISON WITH STATE-OF-THE-ART METHODS

We further compare the results achieved by our TDNet method with those of the state-of-the-art methods using ADHD-200 database for ADHD identification. It is worth noting that all these methods use the standard training/test sets division by the data set. Diagnostic performance of these methods is summarized in Table 1, where the best results are highlighted in bold. From Table 1, we can see that our method can obtain competitive results in nearly all imaging sites for ADHD vs. NC classification. Specifically, our method achieves the average accuracy of 73.2% for ADHD classification, which is higher than the second-best average accuracy value of 72.0% (achieved by (Zhang et al., 2017)) on the same dataset. The results show that considering higher-order interactions and temporal dynamic patterns of FC is helpful in improving diagnosis performance.

REFERENCES

Dey, S., Rao, A. R., and Shah, M. (2012). Exploiting the brain's network structure in identifying ADHD subjects. *Frontiers in Systems Neuroscience* 6, 75

Dey, S., Rao, A. R., and Shah, M. (2014). Attributed graph distance measure for automatic detection of attention deficit hyperactive disordered subjects. *Frontiers in Neural Circuits* 8, 64

Table 1. Comparison with existing studies for ADHD identification using ADHD-200 dataset.

Method	Site					Average
	KKI	NI	NYU	OHSU	PKU	
PCA-LDA Dey et al. (2012)	72.7	**72.0**	70.7	73.5	62.7	70.3
SICE Zhang et al. (2017)	63.6	**72.0**	70.7	79.4	**74.5**	72.0
AGDM Dey et al. (2014)	54.6	48.0	-	**82.4**	58.8	60.9
3D CNN Zou et al. (2017)	72.8	-	70.5	-	63.0	68.8
EM-MI Dou et al. (2020)	**81.8**	-	63.4	-	70.6	71.9
TDNet (Ours)	**81.8**	68.0	**73.2**	76.5	66.7	**73.2**

Dou, C., Zhang, S., Wang, H., Sun, L., Huang, Y., and Yue, W. (2020). ADHD fMRI short-time analysis method for edge computing based on multi-instance learning. *Journal of Systems Architecture* 111, 101834

Zhang, J., Zhou, L., and Wang, L. (2017). Subject-adaptive integration of multiple sice brain networks with different sparsity. *Pattern Recognition* 63, 642–652

Zou, L., Zheng, J., Miao, C., Mckeown, M. J., and Wang, Z. J. (2017). 3D CNN based automatic diagnosis of attention deficit hyperactivity disorder using functional and structural MRI. *IEEE Access* 5, 23626–23636
